# Large second harmonic generation enhancement in Si_3_N_4_ waveguides by all-optically induced quasi-phase-matching

**DOI:** 10.1038/s41467-017-01110-5

**Published:** 2017-10-18

**Authors:** Adrien Billat, Davide Grassani, Martin H. P. Pfeiffer, Svyatoslav Kharitonov, Tobias J. Kippenberg, Camille-Sophie Brès

**Affiliations:** 1 Ecole Polytechnique Fédérale de Lausanne, 1015 Lausanne, Switzerland, Photonic Systems Laboratory (PHOSL), STI-IEL, Station 11, CH-1015 Lausanne, Switzerland; 2 Ecole Polytechnique Fédérale de Lausanne, 1015 Lausanne, Switzerland, Laboratory of Photonics and Quantum Measurements (LPQM), SB-IPHYS, Station 3, CH-1015 Lausanne, Switzerland

## Abstract

Efficient second harmonic generation in integrated platforms is usually achieved by resonant structures, intermodal phase-matching or quasi-phase matching by periodically poling ferroelectric waveguides. However, in all these structures, it is impossible to reconfigure the phase-matching condition in an all-optical way. Here, we demonstrate that a Watt-level laser causes a periodic modification of the second-order susceptibility in a silicon nitride waveguide, allowing for quasi-phase-matching between the pump and second harmonic modes for arbitrary wavelengths inside the erbium band. The grating is long-term inscribed, and leads to a second harmonic generation enhancement of more than 30 dB. We estimate a *χ* ^*(2)*^ on the order of 0.3 pm/V, with a maximum conversion efficiency of 0.05% W^−1^. We explain the observed phenomenon with the coherent photogalvanic effect model, which correctly agrees with the retrieved experimental parameters.

## Abstract (web summary)

Phase-matching and quasi-phase-matching in periodically poled ferroelectrics ensure efficient harmonic generation, but only statically. Here, Billat et al. demonstrate all-optically reconfigurable second harmonic generation by inscribing a stable grating in CMOS-compatible nonlinear waveguides.

## Introduction

Over the last decade, integrated photonics allowed the demonstration of micrometre-scale and low power optical nonlinear devices. In particular, complementary metal–oxide–semiconductor (CMOS) compatible materials such as silicon and silicon nitride (SiN) are the most promising for nonlinear optical signal processing based on third-order processes^1, 2^. However, integrated waveguides showing significant second-order optical nonlinearity are key to enabling a new range of on-chip applications such as self-referencing of chip-based frequency combs^3^, or telecom signal up-conversion in order to perform direct detection with integrated silicon photodiodes^4^. On-chip frequency down-conversion for quantum optics^5^ is also an option, as second-order processes facilitate the pump rejection in photon-pair generation experiments^6^.

SiN exhibits a very large transparency window, from ultraviolet to mid-infrared, and moderate second-order nonlinearity due to interface symmetry breaking, higher multipole bulk terms and a non-isotropic distribution of the SiN dipoles inside the amorphous matrix^7–9^. To enhance second harmonic generation (SHG) in SiN, researchers used resonant structures like microresonators^10^ and waveguide gratings^11^, but at the expense of using fixed operational wavelengths. Moreover, momentum conservation for SHG in integrated waveguides is generally achieved through intermodal phase-matching, implying that the effective index of the pump mode is equal to the second harmonic one, i.e. *n*_p_ = *n*_sh_. Phase-matching is therefore constrained by waveguide design. To overcome this restriction, quasi-phase-matching (QPM) techniques are routinely employed by periodically poling waveguides made of polar materials such as lithium niobate^12^, lithium tantalate^13^ or suitable polymers^14^. However, their integration on silicon photonic circuits still faces limitations^15^. Up to now, none of the presented integrated platforms allows the all-optical and permanent configuration of the phase-matching condition in the waveguide, nor its dynamic update.

Here we demonstrate an optically induced and dynamically reconfigurable SHG enhancement in an integrated photonic platform. We report the growth of the SHG signal over time when pumping a SiN waveguide with a pulsed laser in the communication band (designated as pump). By probing the waveguide with a tunable continuous wave (CW) laser (designated as probe) following the SHG growth, we observe a clear phase-matching peak centred on the pump wavelength. Shifting the pump to another arbitrary wavelength in the 1534–1550 nm range, we notice a similar SHG growth as well as a subsequent phase-matching peak at the new wavelength. Moreover, the waveguide features the same SHG level when probed over a few days, demonstrating a persistent change in the phase-matching condition caused by the pump. Our experimental observations fit well within the framework of the coherent photogalvanic theory^16^, developed to explain the poling occurring in optical fibres illuminated by kilowatt near-infrared lasers^17^.

## Results

### SHG enhancement in the pulsed regime

**Fig. 1:**
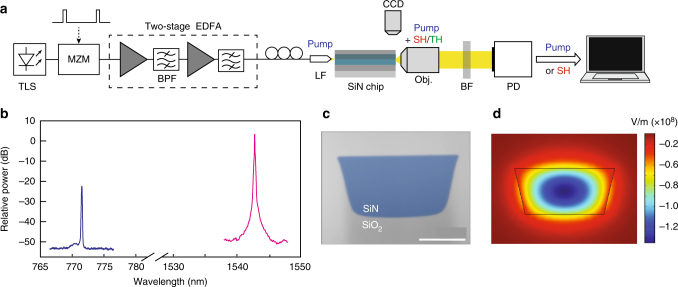
Experimental setup and waveguides. **a** Experimental set-up. TLS, tuneable laser source; MZM, Mach-Zehnder modulator; EDFA, erbium-doped fibre amplifier; BPF, fibre band pass filter; LF, lensed fibre; CCD, digital camera; BF, free-space block filter assembly; PD, power detector; SH, second harmonic; TH, third harmonic. **b** Pump (magenta line) and second harmonic (blue line) spectra at the waveguide output for a coupled peak power of 90 W, after the second harmonic growth. No significant pump broadening is observed. **c** Scanning electron microscope picture of a waveguide cross-section (*scale bar*: 0.5 μm). **d** Simulation of the pump profile, injected on the fundamental transverse magnetic mode

Figure 1a shows the experimental setup, which consists of an amplified CW C-band tunable laser shaped into a pulse train by a Mach-Zehnder modulator. The pulse duration is 200 ps, at a repetition rate of 25 MHz. The modulator can be bypassed to probe SHG with CW light. We couple light to the waveguide fundamental mode with a lensed fibre and evaluate the in-coupling loss to 4.5 dB. At the chip output, a microscope objective collimates the light towards a silicon power detector. In order to attenuate the remaining pump light, as well as the visible light from third-harmonic generation (THG), we placed an assembly of short and long pass filters on the beam path. Out-coupling losses at the second harmonic wavelength are harder to estimate because of higher scattering at shorter wavelengths. We consider a lower boundary of 5 dB including the attenuation coming from the filter assembly. Alternatively, butt coupling the waveguide output to a multimode fibre allows for the measurement of output spectra with an optical spectrum analyser (OSA). An example of pump and second harmonic spectra after enhancement is shown in Fig. 1b, which illustrates the negligible pump broadening through the waveguide.

The waveguides are fabricated according to the photonic Damascene process^18^ (see Methods for fabrication details), which guarantees a crack-free nitride layer deposition and void-free structures of high aspect ratio. The devices are made of stoichiometric Si_3_N_4_ buried in SiO_2_ and exhibit a very low attenuation (0.2 dB/cm). The waveguides are folded in meanders and have trapezoidal cross sections (see Fig. 1c). We obtained results with two different samples: waveguide (i) is 4 cm long and 1.5 μm wide, while waveguide (ii) is 5.8 cm long and 1.4 μm wide. Both are 0.87 μm thick and terminated by an inverse taper mode converter to ease light coupling and reduce Fresnel reflection at the facets.

**Fig. 2:**
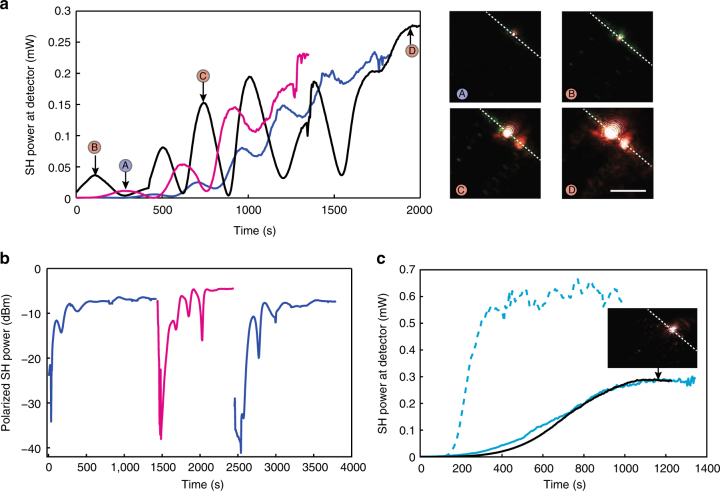
Second harmonic enhancement over time under pulsed pumping. **a** Growth curves of the second harmonic generation (SHG) average power over time in waveguide (i), for pump wavelengths of 1539 nm (magenta line), 1544 nm (blue line) and 1549 nm (black line). The coupled peak power is 90 W. The pictures show the light scattered at the end facet, coming either from second harmonic generation (SHG, red light) or third harmonic generation (THG, green light) The edge of the chip is indicated by the dashed white line (*scale bar*: 20 μm). The labels (A)-(D) indicate the instant and for which pumping wavelength the picture was taken. **b** Power of the transverse electric (TE) or transverse magnetic (TM) SHG component over time when the pump polarization is switched, at the constant wavelength of 1544 nm, from the TE to TM mode, and back to TE in waveguide (i). At each switching point the polarizer is first rotated by 90°, entailing a measured SHG power drop, then the pump polarization is aligned parallel to the polarizer axis, triggering the growth of the second harmonic component. Blue line: power of TE SH under TE pump. Magenta line: power of TM SH under TM pump. **c** SHG growth over time, for pump wavelengths of 1537 (cyan line) and 1550 (black line) nm in waveguide (ii). The coupled peak power is varied between 60 (solid line) and 90 W (dash line). The inset shows the light scattered at the end facet once saturation is reached, with very little green emission

We first injected the pulsed pump in waveguide (i) with 90 W of coupled peak power into the transverse magnetic (TM) mode, which corresponds to an intensity of about 9 GW/cm^2^. The weak initial second harmonic, reaching the detector with an average power of 150 nW, is generated by intermodal phase-matching on a higher order mode. The SHG increased to approximately 200–250 μW average power on the detector (corresponding to 40–50 mW peak) within 25–30 min. In this interval, we kept the pump power constant and observed that any second harmonic generated was TM polarized, as the pump. The SHG growth as a function of time can be triggered by different pump wavelengths, even widely separated, in the same waveguide. This behaviour is illustrated in Fig. 2a for three pump wavelengths within the amplifying band of the erbium-doped fibre amplifier (EDFA), namely 1539, 1544 and 1549 nm. Once saturation of the SHG is reached for a given pump wavelength, changing the pump wavelength results in systematic growth of its second harmonic. In all cases, we observe at saturation more than 30 dB SHG enhancement over time. All the saturation levels are comparable, and slight variations come from non-identical coupling conditions. A visible camera, placed above the chip, images the light scattered out-of-plane at the end facet. Images of this scattered light at different growth points are shown in Fig. 2a. As a function of time, we observed a constant third harmonic generation (THG) (green light) together with an overall growing of the SHG (red light).

Owing to the waveguide birefringence, changing the pump polarization alters the phase mismatch between the pump and the second harmonic, similar to what happens when changing the pump wavelength. To test this behaviour, we alternatively pumped the transverse electric (TE) and TM modes of waveguide (i), keeping the same pump wavelength (1544 nm) and peak power (90 W). At the output of the chip, we measured the SHG component parallel to the pump polarization using a polarizer positioned after the filter assembly. We first pumped the TE mode, and observed the TE second harmonic component growth until saturation. Rotating the polarizer by 90°, we observed that the TM component of the SHG was 30 dB weaker than the TE. Setting the pump to the TM mode, the TM component of the second harmonic grew by three orders of magnitude. After saturation of the TM SHG, the output polarizer was rotated by another 90° transmitting only a negligible TE SHG component. However, switching the pump back to the TE mode systematically re-triggered the SHG growth on the TE polarization, which went back to the initial saturation level in an identical amount of time. Figure 2b shows the corresponding SHG curves. This experiment is a further demonstration that the SHG enhancement can be dynamically updated when the coherence length between the pump and the second harmonic is changed. The slight differences in saturation levels can be explained by the difference in mode overlap in the TE and TM cases.

We then tested waveguide (ii) by pumping in the TE fundamental mode. In this configuration, green light generation is greatly suppressed, as THG is not phase-matched. As for waveguide (i), SHG growth with a pulsed pump is observed. Figure 2c shows that keeping the coupled pump power constant (60 W), we measured similar saturation levels (about 280 μW) and growth durations for different C-band pumping wavelengths (1548 and 1535 nm), as in the case of waveguide (i). Increasing the pump power to 90 W leads to a higher SHG saturation, reaching an average power of 600 μW (120 mW peak) onto the detector, about twice the value reported in waveguide (i). This higher saturation is expected as waveguide (ii) is 45% longer than waveguide (i). However, contrary to waveguide (i), no oscillations in the growth curves were observed and saturation is reached about five times faster.

### Phase-matching evidence in continuous-wave regime

**Fig. 3:**
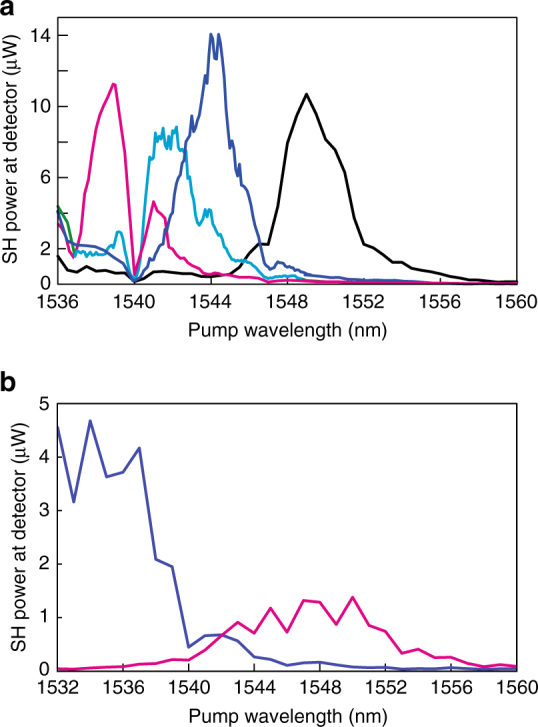
Quasi-phase-matching evidence under continuous-wave probing. **a** Detected second harmonic power as a function of the probe wavelength in waveguide (i) for a continuous-wave (CW) coupled probe power of 350 mW. The waveguide was previously pumped at 1539 nm (magenta line), 1542 nm (cyan line), 1544 nm (blue line) and 1549 nm (black line). Phase-matching peaks are observed around each pump wavelength. **b** Detected second harmonic power as a function of CW probe wavelength for 130 mW of coupled power in waveguide (ii), previously pumped at either 1537 nm and 90 W coupled power (blue line) or 1550 nm and 60 W coupled power (magenta line)

After SHG saturation, we probed the waveguides with a CW tunable laser, keeping the coupled probe power constant at 350 mW. Under such light exposure, we did not notice any SHG evolution over time. We plot the second harmonic power as a function of wavelength in Fig. 3a. We probed waveguide (i) after four pump wavelengths: 1539, 1542, 1544 and 1549 nm. In all cases, one notices a clear peak near the pulsed pump wavelength. This is an indication that the strong pulsed pump modulates the waveguide second-order nonlinearity with the correct periodicity to quasi-phase match the SHG. Therefore, the resulting *χ* ^*(2)*^ grating derives from an all-optical poling mechanism. This QPM condition is fulfilled every time the pump wavelength is changed, in spite of the waveguide dispersion. The peak 3 dB bandwidth (FWHM) is about 2.5 nm (at the pump wavelength) while their maxima are located 10 dB higher than what can be considered as the floor level for SHG.

We also probed waveguide (ii) by coupling a 130 mW CW laser, after pumping at 1535 nm with 90 W peak power and at 1548 nm with 60 W peak power. We again observed phase-matching peaks around the pump wavelengths (see Fig. 3b). In addition, the power ratio of the SHG between the two cases is comparable to that of the saturation powers in the pulsed regime, as can be seen in Fig. 2c.

### Microscopic model based on the coherent photogalvanic effect

The effect reported here is qualitatively similar to the all-optical SHG enhancement reported in silica fibres pumped by a kilowatt-level pulsed Nd:YAG laser^17^. Researchers identified the coherent photogalvanic effect (CPE) as the main underlying mechanism^16, 19^. Optical poling has also been reported in polymer fibers^20, 21^, however in that case the physical origin of the poling has been ascribed to an orientation mechanism of the molecules inside the polymer matrix, with no apparent contribution from a space-charge field^20^.

**Fig. 4:**
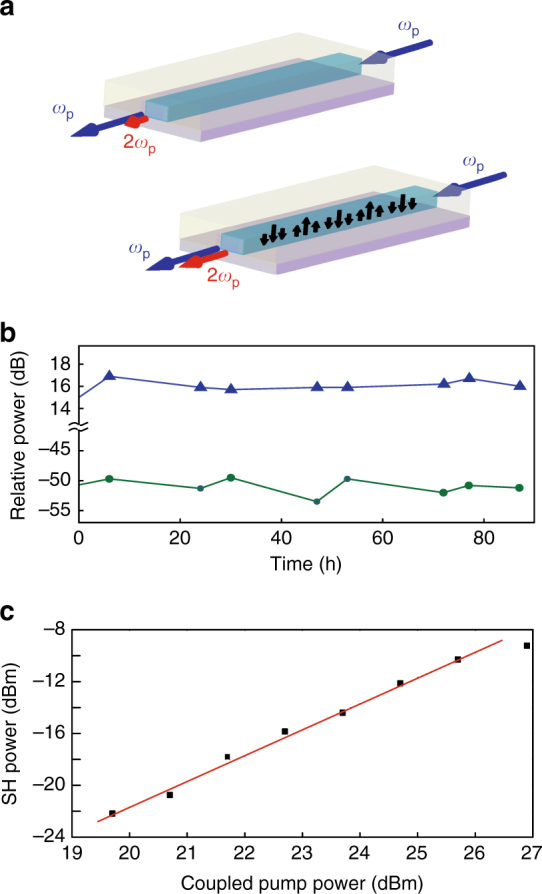
Grating dynamics and evaluation of its parameters. **a** Illustration of the *χ* ^*(2)*^ grating inscription in a SiN waveguide. After irradiation, a spatially periodic DC field builds up with a periodicity that is twice the coherence length between the pump and second harmonic mode. *ω*_p_ indicates the pump frequency. **b** Persistence measurement showing the continuous wave probe (blue triangles) and second harmonic (green circles) power over more than 80 h of operation. Both quantities were measured at the waveguide output. **c** Second harmonic power (estimated in waveguide (i)) as a function of the coupled continuous wave probe power. The probe is centred at 1544 nm, and the grating was previously inscribed at the same wavelength. The squares are experimental points while the red line is a linear fit with a slope of 2. The observed saturation at high power comes from coupling instabilities

The CPE consists in an asymmetric photoemission of electrons from defect centres with energy levels lying inside the band gap of the material (such as GeO_2_-related defects in silica). It takes place under illumination by a pump and its frequency-doubled counterpart, as the result of the interference from different coherent multiphoton absorption processes involving the two waves. The spatially preferential emission of electrons gives rise to a photogalvanic current (see Supplementary Note 1) which in turns builds up a static space-charge field (*E*_DC_)^22^. As schematically illustrated in Fig. 4a, the weak initial SHG together with the strong pump, triggers the increase over time of *E*_DC_ by the CPE process. *E*_DC_ has a periodicity that directly depends on the coherence length between the pump and its second harmonic^16^. The effective *χ* ^*(2)*^, which allows QPM of the SHG at the pump wavelength^23^, thus arises as the product of *χ* ^*(3)*^ and the built-in field, a process known as electric field induced second harmonic generation (EFISHG)^24^. The gratings are long-term inscribed. In fact, the ejected electrons, responsible for the built-in *E*_DC,_ are eventually trapped by deep and localized defect states^19^.

The Si-Si defects in deposited SiN, extensively studied in the frame of electronic memories^25–27^, are good candidates to be responsible for this trapping effect. According to these previous works, localized defect energy levels lie inside the ~4.6 eV bandgap at about 1.4 and 3.2 eV from the valence band. These localized states can act as electron emission sites and as long-lived electron traps, and their energy location agrees well with the CPE theory, for a pump laser at 1.55 μm (see Supplementary Note 1 for more details). We verified the persistence of the SHG by probing the sample over multiple days. As seen in Fig. 4b, no decrease in SHG efficiency was observed such that the grating is indeed long-term inscribed. This model is also in agreement with the adverse impact of THG on the SHG growth, as observed in waveguide (i). In fact, green photons with a ~2.4 eV energy are able to directly re-ionize the electrons trapped in acceptor states below the conduction band^28^. Such a linear absorption process does not lead to any preferential emission and the electrons just move accordingly to the E_DC_, opposite to the direction of the photogalvanic current. Moreover, CPE can also involve multiphoton coherent interference from one pump photon and one photon at the THG frequency, albeit with the wrong periodicity. We therefore ascribe the oscillations observed during the growth of SHG in waveguide (i) to the counter-acting effects of CPE involving photons from THG and direct re-ionization also due to third harmonic photons.

We verified the quadratic relationship between the CW probe power and the second harmonic power at the phase-matching peak in the 1544 nm pump case, as shown in Fig. 4c. The retrieved conversion efficiency is 0.05% W^−1^, which corresponds to about 3∙10^−3^% W^−1^ cm^−2^. Assuming quasi-phase-matched SHG with a sine-modulated *χ* ^*(2)*^ grating^29^, we can also estimate the amplitude of the *χ* ^*(2)*^ in waveguide (i) by fitting the data from Fig. 4c with Eq. (1). In this equation *ω*_sh_ is the second harmonic frequency, *P*_p_ the coupled pump power, *L* the waveguide length and *S* the overlap integral between the pump and second harmonic mode.

1 $${P_{sh}} = {\left( {\frac{{2{\omega _{sh}}{\chi ^{(2)}}{P_p}LS}}{{\pi c{n_{sh}}}}} \right)^{\!\!\! 2}}$$

With the help of a mode solver, we compute the effective indices and the mode overlap of the TM modes at the pump and SHG frequencies in waveguide (i). We assume that the SHG occurs on the TM 8th dipolar mode (modes are numbered according to their effective index), which features the best phase-matching and mode overlap with the pump. Indeed, since the natural initial SHG takes place on a higher order mode thanks to intermodal phase-matching^10^, the QPM grating period is twice the initial coherence length. From the simulated mode profiles, we estimate a peak *χ* ^*(2)*^ value of about 0.3 pm/V, and we calculate the grating period to be *Λ* = 2*π*/|*β*_sh_ − 2*β*_p_| ≈ 43 μm (where *β* is the mode propagation constant). For waveguide (ii), the SHG is expected to happen on the 8^th^ TE mode. In this case, we retrieve from Fig. 3b that *χ* ^*(2)*^ ≈ 0.15 pm/V, consistent with the estimation in waveguide (i), and a grating period of about 64 μm at 1535 nm. As the second harmonic always has the same polarization as the pump, these values refer to the diagonal *χ* ^*(2)*^ tensor element. All the mode simulations and calculations are detailed in Supplementary Note 2. Then, via the relation *χ* ^*(2)*^ = 3*χ* ^*(3)*^ *E*_DC_^19^, we calculate a space-charge field magnitude of 10^8^ V/m, assuming a third-order susceptibility of 10^−21^ m^2^/V^2^ ^30^. Finally, it has to be noted that because of intermodal phase-matching, the FWHM of the SH depends on the difference between the slopes of the fundamental and the second harmonic effective index curves, at the second harmonic wavelength. According to Supplementary Fig. 2, the expected linewidth should be less than 1 nm for both waveguide (i) and (ii). This value is smaller than the FWHM reported in Fig. 3 for the tested waveguides (few nanometers). A possible explanation could lie in a temperature gradient over the waveguides’ length. In fact, despite the fast (microsecond range) thermal relaxation time due to the thermo-optic effect in SiN waveguides^30^, different optical absorptions at different waveguide’s positions would lead to a deviation of the coherence length between the pump and the second harmonic along the waveguide. This coherence length distribution is equivalent to a chirping of the QPM grating, known to broaden the SHG conversion efficiency.

## Discussion

We have experimentally demonstrated an all-optical and reconfigurable SHG enhancement by more than 30 dB in a popular SiN waveguide platform, ready to be fabricated on a large scale in multi-project wafer runs. The enhancement results from the persistent inscription of a second-order susceptibility grating in the waveguide. The grating period automatically adapts to a modified coherence length between the pump and the harmonic, allowing for quasi-phase-matched SHG over the whole C-band. The measured conversion efficiency of 0.05% W^−1^ (3∙10^−3^% W^−1^ cm^−2^) is comparable to the one obtained in similar SiN waveguides designed to fulfil the intermodal phase-matching condition at telecom wavelengths^31^, or in SiN ring resonators^10^. This shows the effectiveness of the reported all-optical QPM method. The *χ* ^*(2)*^ value of few tenths of pm V^−1^ is also in agreement with previous measurements in SiN waveguides at the same wavelengths^31^. The estimated DC field magnitude, assuming the coherent photogalvanic effect as the underlying physical mechanism, is comparable to the one obtained in ref. ^31^, where a voltage is applied across the waveguide structure to enhance SHG.

In parallel, silicon waveguides recently achieved remarkable SHG efficiencies, either thanks to a SiN strain layers^32^ or by leveraging the EFISHG through a p-i-n junction created by ion implantation^33^. The presented findings however feature an improved and appealing versatility compared to the current strategies for on-chip integration of second-order nonlinearity. In fact, they show a unique method to configure dynamically QPM in passive devices fabricated with CMOS compatible steps.

Compared to previous works in silica optical fibres, the use of integrated waveguides allows to increase the conversion efficiency by two orders of magnitude, and to decrease by the same factor the SHG growth duration^17^. Various options for improving the conversion efficiencies can be explored. As shown here, the efficiency of the process increases with the length of the waveguide and the pump power. In addition, the saturation of the CPE is given by the pump-second harmonic mode overlap, the density of the defect states and the *χ* ^*(3)*^ value. Simulations indicate that seeding the process with a second harmonic wave injected on the fundamental mode, in order to improve the mode overlap, may increase the SHG efficiency by 40%. Moreover, the low propagation loss (0.2 dB/cm) at the pump wavelength results in a long effective length (about 22 cm), such that longer waveguides can be considered for enhanced conversion efficiency. Employing materials with a larger *χ* ^*(3)*^, such as Si-rich SiN^34^, is also an option to increase the SHG by at least an order of magnitude^35^.

## Methods

### Waveguide fabrication

The waveguide devices were fabricated using the photonic Damascene process^18^. The process starts by patterning the waveguides as well as a dense filler pattern into a hard mask of amorphous silicon on a 4 μm thick wet thermal silicon oxide. The structures are then transferred into the preform using a dry etch process based on He and C_4_F_8_. Next the waveguide trenches in the preform are filled with low pressure chemical vapour deposition silicon nitride, deposited in one run up to the desired thickness. The dense filler pattern efficiently releases film stress and prevents cracking of the SiN thin film. The excess SiN is removed using chemical mechanical polishing, providing a smooth and planar wafer surface. Finally, the wafer is annealed to drive out residual hydrogen in the films (1200 °C, 24 h, N_2_ atmosphere) and cladded with low temperature oxide (LTO), before being separated into individual chips.

### Data availability

The data that support the findings of this study are available from the authors on reasonable request, see author contributions for specific data sets.

## Electronic supplementary material

Supplementary Information
