# Supplementary material for: Large second harmonic generation enhancement in Si3N4 waveguides by all-optically induced quasi-phase-matching
Source: Nat Commun. 2017 Oct 18;8:1016. doi: 10.1038/s41467-017-01110-5 (PMC5725590; doi:10.1038/s41467-017-01110-5)
Supplement: Supplementary file 1 — Supplementary Information [file 41467_2017_1110_MOESM1_ESM.pdf]

Supplementary Note 1: coherent photogalvanic effect in silicon nitride

The photogalvanic effect corresponds to the appearance of a current in a non-centrosymmetric medium under uniform illumination. In fibres, researchers postulated that asymmetric photoemission takes place from defect centres (such as GeO_2-related defects in silica) under illumination by a pump and its second harmonic, as a result from the coherent interference between different multiphoton absorption processes, resulting in a coherent photogalvanic effect (CPE). In particular, this model was developed considering the interference between a three-photon absorption and both the two- and four-photon absorption[1,2]. In a one-dimensional approximation, the algebraic coherent photogalvanic current j_{ph} has a dependence given by the equation below[1].

$$j_{\mathrm{ph}} \propto i * \eta_1 |E_{\mathrm{p}}|^2 |E_{\mathrm{sh}}| \left(\eta_2 |E_{\mathrm{p}}|^4 - |E_{\mathrm{sh}}|^2\right) \exp\left(i(\Phi_{\mathrm{sh}} - 2\Phi_{\mathrm{p}})\right) + \mathrm{c.c.} \quad (1)$$

where E is the local electric field of the pump or second harmonic (SH) (denoted by the subscript p or sh, respectively), Φ is the phase of the corresponding fields (same subscript), η_1 and η_2 are coefficients of multi-photon absorption, and c.c. indicates the complex conjugate. The exponential term denotes the coherent nature of the process, and leads to spatially periodic charge separation. Charges therefore migrate in a preferential direction and j_{ph} gets associated to a space-charge DC electric field E_{DC}, whose periodicity allows for quasi phase matching the second harmonic generation (SHG). The relation between the two is given by the photoconductivity σ, proportional to the total density of carriers promoted to the conduction band. This relation is $j_{\mathrm{ph}} = \frac{E_{\mathrm{DC}}}{\sigma}$. For a certain level of displaced carriers, E_{DC} reaches a threshold value that prevents a further migration of photo-carriers, leading to the saturation of the process. Finally, the trapping of displaced carriers by deep defect states near the conduction band explains the space-charge field persistence.

In the case of SiN, nitrogen vacancies saturated with hydrogen atoms, leading to a Si–H bond and a covalent Si-Si bond, are good candidates to act as traps for both holes and electrons. Most of the

previous works agree in placing the electron and hole trap energies inside the ~4.6 eV bandgap of SiN at around 1.4 eV from the conduction and valence band, respectively[3,4.] Therefore, electron promotion from an occupied defect state to the conduction band requires roughly 3.2 eV. The photon energy for a 1.55 µm pump is 0.8 eV, while the SH photon energy is 1.6 eV. The interferential multi-photon absorption process described in Supplementary Equation 1 considers absorption processes exactly corresponding to a total energy of 3.2 eV, showing that these Si-Si defects are good candidates to explain asymmetric carrier ejection. Nevertheless, electron trap energies (W_t^e) have been measured in the range 1.2 – 1.7 eV below the conduction band, while hole traps lie within W_t^h = 0.9 – 1.7 eV above the valence band[3,4,5,6] (see Supplementary Figure 1). Moreover, some variation in the energy traps has also to be considered when moving from neutral to positively charged donor (negatively charged acceptor) defects[4]. Considering these ranges, a two-step process cannot be excluded. In this case, an electron from the donor level is promoted to the acceptor one by one SH photon or by two pump photons. Subsequently, a first order CPE process involving the interference between the two pump photons and the one SH photon absorptions would take place giving the correct coherent phase term for quasi-phase matching (QPM): $\Phi_{sh} - 2\Phi_p$.

Moreover, SiO_2-SiN structures can localize electrons and holes for multi-year lifetimes[7]. Since these Si-Si defects also entail localized states ~1.4 eV below the conduction band, they can potentially trap free carriers as well. It would result in a long-lived and thermalization-immune localization of the photo-carriers, in agreement with the observed grating persistence.

In our experiments, we also observed the adverse effect of green light from third harmonic generation (THG) on the SH enhancement dynamics, manifesting itself for instance by oscillations in the growth curves in waveguide (i). The erasure of $\chi^{(2)}$ gratings by visible radiation was reported in fibres as well[8]. If the electrons are promoted from defects to the conduction band via single-photon absorption

(without any interference process), there is no preferential ejection direction on average. They are thus subject to the built-in electric field only and entail a drift current j_{d} opposite to the photogalvanic one. In our case, green light has energy of 2.4 eV, sufficient to excite the carriers trapped in defect states near the conduction band and lead to their recombination, and to the grating erasure. Moreover, THG, as well as SHG, is a coherent process. Thus, in the presence of strong THG, the CPE can involve a coherent interference between the absorption of one pump photon and one third harmonic (TH) photon. However, this process entails a grating period inversely proportional to $|\beta_{\mathrm{th}} - \beta_p|$, being β_{th} the propagation constant of the TH field, counteracting the growth of the grating with the right periodicity for QPM of the SHG.

Supplementary Note 2: Evaluation of $\chi^{(2)}$ after grating inscription

The overlap integral *S* between the pump and SH mode, appearing in Equation 1 of the main text, has an analytical formula derived from the coupled mode theory[9] and given by the equation below.

$$S = \iint_A E_{\mathrm{p}}^2(\mathrm{x},\mathrm{y}) E_{\mathrm{sh}}(\mathrm{x},\mathrm{y}) \,\mathrm{sign}(\chi^{(2)}) dxy \qquad (2)$$

In this formula, the integration is carried out over the entire waveguide cross section. The electric fields are normalized to the power flux (P_{z}) across the waveguide cross section given by the mode solver: $\iint_A |E_i|^2 dxy = \frac{2P_{\mathrm{z}}}{c\varepsilon_0 n}$, where *i* = *x or y*, depending on the field polarization.

The local value of the space charge field E_{DC} has an orientation and a magnitude that depends upon the phase difference between the pump and SH. The $\chi^{(2)}$ grating, which originates from this field via the third-order susceptibility tensor ($\chi^{(2)} = 3\chi^{(3)}E_{\mathrm{DC}}$), also follows this rule. In fact, this $\chi^{(2)}$ dependence holds whatever is the microscopic model behind the phenomenon, as verified in the main text by measuring the QPM peaks. Yet, in our waveguides, the SH propagates on a dipolar or tripolar mode (see Supplementary Figure 2). The electric field in each lobe is in quadrature with the field in the

adjacent lobe(s). On the contrary, the pump field in the fundamental mode has a constant phase over the waveguide cross section. The space charge field E_{DC} must therefore have opposite signs in each lobe region. Consequent, $\chi^{(2)}$ also switches sign from one lobe to the other, as does the SH electric field phasor. The overlap integral can thus be rewritten as $S = \iint_A E_{\mathrm{p}}^2(\mathrm{x},\mathrm{y}) \left| E_{\mathrm{sh}}(\mathrm{x},\mathrm{y}) \right| dxy$.

We performed the evaluation of $\chi^{(2)}$ and of grating period by computing the waveguide dispersion and mode profile with a finite-element solver (COMSOL Multiphysics). In all cases, the SH is assumed to propagate on the high order mode that yields the smallest index difference (i.e. which yields the best natural phase matching) and the best mode overlap with the pump. QPM then occurs via CPE to compensate for the index mismatch. In waveguide (i), only the transverse magnetic (TM) modes are considered (Supplementary Figure 2a), since both the pump and harmonic are TM when probed in CW. The SH mode featuring the smallest index difference and largest mode overlap with the pump is the 8th TM mode. Supplementary Figure 2b and c shows the simulated mode profile of the pump and SH mode of waveguide (i). In the case of waveguide (ii), only the transverse electric (TE) modes are considered and their index curves are displayed in Supplementary Figure 2d. The pump propagates over the fundamental TE mode (see Supplementary Figure 2e), while the second harmonic is in the 8th TE mode, with a profile reported in Supplementary Figure 2f.

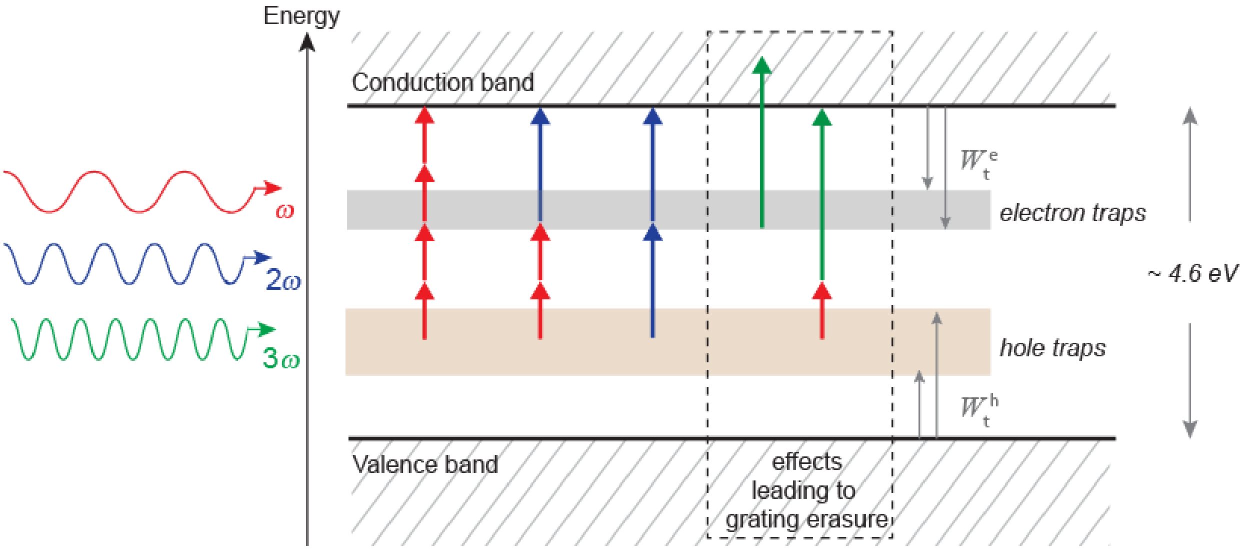

Supplementary Figure 1: energy diagram for the coherent photogalvanic effect in silicon nitride. Scheme of the energy diagram of the defect states, coming from nitrogen vacancies, inside the silicon nitride (SiN) band gap. Red arrows indicate the energy of the pump photons, blue and green arrows represent the energy of the second and third harmonic photons, respectively. High order coherent photogalvanic effect (CPE) can directly promote electrons from donor levels (lower energy defects) to the conduction band. Otherwise, a two-step process involving first the electron promotion to the acceptor levels (higher energy defects) and then a first order CPE to the conduction band is also possible. Given the range for the energy of the electron trap, third harmonic photons can directly ionize these defect states and promote electrons to the conduction band. Moreover, a process involving one pump photon and one third harmonic photon can also participate to a high order CPE, leading however to the wrong grating periodicity for quasi-phase matching the second harmonic generation.

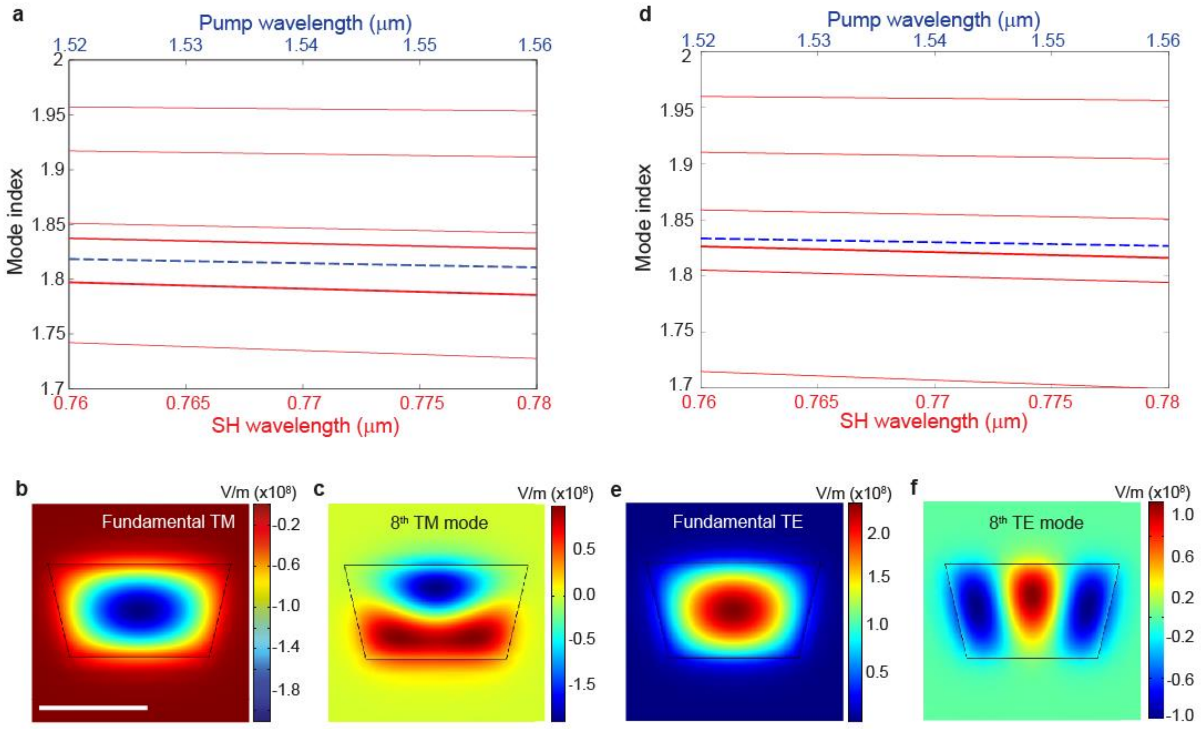

Supplementary Figure 2: numerical simulation of the waveguide modes. a, Effective index curves for the transverse magnetic (TM) modes in waveguide (i), as computed with a mode solver. The solid red curves correspond to higher order modes at the second harmonic wavelength (bottom x axis), while the dashed blue curve corresponds to the fundamental mode at the pump wavelength (top x axis). **b,** Simulated profile for the fundamental TM mode at 1540 nm in (i). **c,** Simulated profile for the 8th TM mode at 770 nm in (i). This mode is right above the fundamental one. **d,** Effective index curves for the transverse electric (TE) modes in waveguide (ii). **e,** Simulated profile for the fundamental TE mode at 1540 nm in (ii). **f,** Simulated profile for the 8th TE mode at 770 nm in (ii). This mode is right below the fundamental one. (scale bar: 1 μm).

Supplementary References

1. Anderson, D. Z., Mizrahi, V., Sipe, J. E. & Ban, C. Model for second-harmonic generation in glass optical fibers based on asymmetric photoelectron emission from defect sites. *Opt. Lett.* **16,** 796–798 (1991).

2. Dianov, E. M. & Starodubov, D. S. Photoinduced generation of the second harmonic in centrosymmetric media. *Quantum Electron.* **25,** 395–407 (1995).

3. Gritsenko, V. A., Perevalov, T. V., Orlov, O. M. & Krasnikov, G. Y. Nature of traps responsible for the memory effect in silicon nitride. *Appl. Phys. Lett.* **109,** 062904-1–062904-4 (2016).

4. Vianello, E., Driussi, F., Blaise, P., Palestri, P., Esseni, D., Perniola, L., Molas, G., De Salvo, B., Selmi, L. Explanation of the Charge Trapping Properties of Silicon Nitride Storage Layers for NVMs—Part II: Atomistic and Electrical Modeling. IEEE Transactions on Electron Devices. **8**, 2490-2499 (2011).

5. Vianello, E., Driussi, F., Arreghini, A., Palestri, P., Esseni, D., Selmi, L., N. Akil, Van Duuren, M., Golubovic, D. Experimental and simulation analysis of program/retention transients in silicon nitride-based NVM cells. IEEE Trans. Electron Devices, **56**, 1980–1990 (2009).

6. Naich, M., Rosenman, G., Roizin, Y., Molotskii, M. Exoelectron emission studies on trap spectrum in ultrathin amorphous Si3N4 films. Solid State Electron., **48**, 477–482 (2004).

7. Gritsenko, V. A. *et al.* Excess silicon at the silicon nitride/thermal oxide interface in oxide–nitride–oxide structures. *J. Appl. Phys.* **86,** 3234–3240 (1999).

8. Ouellette, F., Hill, K. O. & Johnson, D. C. Light-induced erasure of self-organized χ^(2) gratings in optical fibers. *Opt. Lett.* **13,** 515–517 (1988).

9. Yariv, A. Coupled-Mode theory for guided-wave optics. *IEEE J. Quantum Electron.* **9,** 919–933 (1973).
